# Profiling and Functional Analysis of Circular RNAs in Porcine Fast and Slow Muscles

**DOI:** 10.3389/fcell.2020.00322

**Published:** 2020-05-26

**Authors:** Bojiang Li, Di Yin, Pinghua Li, Zengkai Zhang, Xiying Zhang, Hongqiang Li, Rongyang Li, Liming Hou, Honglin Liu, Wangjun Wu

**Affiliations:** ^1^Department of Animal Genetics, Breeding and Reproduction, College of Animal Science and Technology, Nanjing Agricultural University, Nanjing, China; ^2^College of Animal Science and Veterinary Medicine, Shenyang Agricultural University, Shenyang, China; ^3^College of Agronomy and Biotechnology, Hebei Normal University of Science and Technology, Qinhuangdao, China

**Keywords:** circular RNA, expression profile, fast-twitch biceps femoris muscle, slow-twitch soleus muscle, pig

## Abstract

The different skeletal muscle fiber types exhibit distinctively different physiological and metabolic properties, and have been linked to both human metabolic diseases and meat quality traits in livestock. Circular RNAs (circRNAs) are a new class of endogenous RNA regulating gene expression, but regulatory mechanisms of skeletal muscle fibers involved in circRNAs remain poorly understood. Here, we constructed circRNA expression profiles of three fast-twitch biceps femoris (Bf) and three slow-twitch soleus (Sol) muscles in pigs using RNA-seq and identified 16,342 distinct circRNA candidates. Notably, 242 differentially expressed (DE) circRNAs between Bf and Sol muscles were identified, including 105 upregulated and 137 downregulated circRNAs, and are thus potential candidates for the regulation of skeletal muscle fiber conversion. Moreover, Gene Ontology (GO) and Kyoto Encyclopedia of Genes and Genomes (KEGG) pathway enrichment analysis of host genes of DE circRNAs revealed that host genes were mainly involved in skeletal muscle fiber-related GO terms (e.g., muscle contraction, contractile fiber part, and *Z* disk) and skeletal muscle fiber-related signaling pathways (e.g., AMPK and cGMP-PKG). We also constructed co-expression networks of DE circRNA-miRNA-mRNA using previously acquired high-throughput sequencing mRNA and miRNA data, from which 112 circRNA-miRNA and 95 miRNA-mRNA interactions were identified. Multiple circRNAs essentially serve as a sponge for miR-499-5p, which is preferentially expressed in slow-twitch muscle and reduces the severity of Duchenne muscular dystrophy (DMD). Taken together, a series of novel candidate circRNAs involved in the growth and development of porcine skeletal muscle was identified. Furthermore, they provide a comprehensive circRNA resource for further in-depth research on the regulatory mechanisms of circRNA in the formation of skeletal muscle fiber, and may provide insights into human skeletal muscle diseases.

## Introduction

Mammalian skeletal muscles with different fiber types exhibit various structural and functional properties. Slow-twitch fibers enrich mitochondria and have high oxidative capacity, and primarily express myosin heavy chain I (MyHC I) (Schiaffino and Reggiani, 2011). In contrast, fast-twitch fibers primarily express myosin heavy chain II b (IIb) and display strong glycolytic metabolic capacity (Schiaffino and Reggiani, 2011). Muscle fiber types are associated with various disorders and diseases, including muscle dystrophy, muscle atrophy, cardiomyopathic disease, type 2 diabetes, and other metabolic diseases (Chakkalakal et al., 2004; Bassel-Duby and Olson, 2006; Schiaffino and Reggiani, 2011; Reyes et al., 2015; Boyer et al., 2019). It has been reported that insulin-stimulated glucose transport is more efficient in slow-twitch muscle than in fast-twitch muscle, and that the calcineurin/PGC-1 pathway enhances slow-twitch fiber abundance, increases insulin-mediated glucose uptake, and improves resistance to diet-induced glucose intolerance (Ryder et al., 2003). Loss of prospero-related homeobox factor 1 (Prox1), a critical transcription factor regulating the conversion of fast-twitch to slow-twitch muscle, results in severe dilated cardiomyopathy (Petchey et al., 2014). Since slow-twitch fibers contain more mitochondria than fast-twitch fibers and predominantly mediate fatty acid oxidation for energy production, increasing the proportion of type I fibers could subsequently improve protection against metabolic diseases, such as obesity (Stuart et al., 2013). As peroxisome-proliferator-activated receptor-γ coactivator-1 (PGC-1α) is a principal factor regulating slow-twitch fiber determination, muscle-specific overexpression of PGC-1α improves the parameters characteristic of Duchenne muscular dystrophy (DMD), a regulatory mechanism mediated by folliculin interacting protein-1 (Fnip 1) (Handschin et al., 2007; Reyes et al., 2015). During postnatal development and regeneration, the muscle fiber types can be modified by controlling the slow or fast muscle gene programs evoked by many external factors, such as exercise and thyroid hormone, and followed the rule of type I ↔ type IIa ↔ type IIx ↔ type IIb (Schiaffino et al., 2007). For example, thyroid hormone induces a fiber-type shift from MyHC I to IIa, IIx, and IIb via miR-133a/TEAD1 (Zhang D. et al., 2014); fast-to-slow fiber-type switch (IIb → IIx → IIa → I) induced by ERK1/2 signaling reduces muscular dystrophy disease severity (Boyer et al., 2019). In livestock, skeletal muscles composed of different fiber types vary in postmortem metabolic rates, and are therefore closely linked with color, water-holding capacity, marbling, pH and other meat quality traits (Hwang et al., 2010). For example, evidence suggests that an increase in the number of IIb fiber lowers muscle pH and enhances muscle cooking loss and lightness (Kim et al., 2013). A better understanding of the genetics basis of muscle fiber formation could therefore greatly improve both meat quality traits of livestock and treatment of human muscle diseases.

Circular RNAs (circRNAs) are essentially closed RNA transcripts that are generated from precursor mRNA back-splicing with covalent linkage at the 3′ and 5′ ends. They differ from their linear counterparts through the absence of a 5′ cap and a polyadenylated [poly(A)] tail (Vicens and Westhof, 2014). CircRNAs are highly conserved in expression patterns and sequences among species (Rybak-Wolf et al., 2015) and, due to their non-linear structure, have greater stability than linear RNAs (Li X. et al., 2018). They are widely expressed in numerous types of human organs, including brain and heart tissues (Rybak-Wolf et al., 2015; Tan et al., 2017). But circRNAs are also associated with several types of disorders, including cancer (Li et al., 2017; Zhang et al., 2018), and diseases of the cardiac (Khan et al., 2016) and skeletal muscles (Legnini et al., 2017). Previous research has shown that circ-QKI and circ-BNC2 are upregulated during *in vitro* differentiation of control myoblasts, and downregulated in DMD conditions (Legnini et al., 2017). Many circRNAs have important biological functions by acting as microRNA or protein inhibitors (“sponges”), or are themselves translated during muscle development and growth (Legnini et al., 2017; Wei et al., 2017; Ouyang et al., 2018; Hong et al., 2019; Kristensen et al., 2019). CircLMO7, a circular RNA identified in bovine skeletal muscle at two developmental stages, was shown to regulate myoblast differentiation and survival by sponging miR-378a-3p (Wei et al., 2017). Similarly, research on chickens has indicated that circSVIL promotes myoblast proliferation and differentiation by acting as a miR-203 sponge (Ouyang et al., 2018). Legnini et al. (2017) found that a circRNA translated into a protein, circ-ZNF609, which is downregulated during myogenesis and regulates myoblast proliferation. However, what roles circRNA may play in the regulation of skeletal muscle fibers in the mammal is still largely unknown.

We previously obtained expression profiles for the coding genes of, and identified the DE genes between, porcine fast-twitch biceps femoris (Bf) and slow-twitch soleus (Sol) muscles using RNA-seq (Li et al., 2016). However, the expression profiles of circRNAs in Bf and Sol muscles and the potential regulatory mechanisms in skeletal muscle fiber types are still unclear. Here we determined the expression profiles of circRNAs and identified the DE circRNAs in Bf and Sol muscles, and performed GO and KEGG enrichment analysis using the host genes of DE circRNAs. We also constructed the circRNA-miRNA-mRNA regulatory network affecting skeletal muscle fiber formation using DE circRNA, miRNAs and mRNAs, then validated circRNA and miRNA binding via dual-luciferase assay. Our results represent a solid basis for further in-depth study of the regulatory mechanisms controlling skeletal muscle growth and development, and the formation of skeletal muscle fiber type mediated by circRNAs in pig. In addition, because muscle fiber types have been linked to many diseases, our data could further inform the development of treatment for human muscular diseases.

## Materials and Methods

### Ethics Statement

All experimental procedures were conducted according to the guidelines of the regional Animal Ethics Committee and approved by the Institutional Animal Care and Use Committee of Nanjing Agricultural University.

### Experimental Animals and Sampling

Pigs used in our experiments derived from the 48 Duroc × Meishan hybrid pig population described previously by Li et al. (2016). All pigs were raised under standard conditions and fed *ad libitum*, and given free access to water. To improve reliability and reduce inter-individual differences, three full-sib female pigs (Samples 28, 35, and 36) with similar growth and carcass traits were selected, following methods described previously (Li et al., 2016). The two kinds of skeletal muscles (i.e., Bf, fast-twitch muscle, and Sol, slow-twitch muscle) were collected from each pig after slaughter, and thus a total of six muscle group (Bf28, Bf35, Bf36, Sol28, Sol35, and Sol36) were included in RNA-Seq. Muscle tissue samples were snap-frozen in liquid nitrogen immediately following collection and stored at −80°C until RNA isolation was performed.

### RNA Isolation, Library Preparation, and Sequencing

Total RNA was extracted from the three Bf and Sol muscles using Trizol reagent (Invitrogen, Life Technologies, Carlsbad, CA, United States) in accordance with the manufacturers’ instructions. RNA purity was measured using a NanoDrop 2000 (NanoDrop Technologies, Wilmington, DE, United States), and RNA integrity and concentration were determined with an Agilent 2100 Bioanalyzer (Agilent Technologies, Santa Clara, CA, United States). Ribosomal RNA (rRNA) was removed using a Ribo-Zero Magnetic Gold Kit (Epicentre, Madison, WI, United States). Subsequently, the six cDNA libraries were prepared with an NEB Next Ultra Directional RNA Library Prep Kit for Illumina (NEB, Ipswich, MA, United States) in accordance with the manufacturer’s instructions. The libraries were then sequenced on a HiSeqXten platform with 150 bp paired-end reads (Illumina, San Diego, CA, United States).

### Identification of DE CircRNA

Low-quality reads (defined as the percentage of the quality value of low-quality bases ≤19 is more than 50% in a read), polluted adapters, high content of unknown bases (defined as N is more than 5% in a read), and the rRNA-mapped reads from the raw data were all removed from the analysis to obtain high-quality clean reads. At the same time, Q30 of the clean data was calculated. Clean reads were then aligned to the pig reference genome (Sscrofa 11.1) using the Burrows-Wheeler Aligner (BWA)-MEM (Li and Durbin, 2009) with default parameters and CIRI (Gao et al., 2015), an efficient and unbiased algorithm for *de novo* circRNA identification from high-throughput transcriptome data, was used to identify circRNAs. Finally, circRNA expression was denoted to spliced reads per billion mapping (SRPBM) using the following formula: SRPBM = (number of back-spliced junction reads)/(number of mapped reads) × 1,000,000,000. DEseq2 (Love et al., 2014) was used to identify the DE circRNAs between the Bf and Sol muscles. A fold change (FC) of ≥2 or ≤0.5 and a Benjamini-Hochberg method corrected *P*-value of <0.05 were considered to indicate significantly DE circRNAs.

### GO and Pathway Enrichment Analysis

Gene Ontology analysis for host genes of DE circRNAs by Gene Ontology terms^1^ was conducted using the Blast2GO program^2^ (Conesa et al., 2005) with an *E*-value cut-off at 10^–5^. Pathway functional annotation for host genes of DE circRNAs was performed through sequence comparisons against the Kyoto Encyclopedia of Genes and Genomes (KEGG) database (Kanehisa Laboratories, Kyoto, Japan)^3^ using BLASTX algorithm (*E*-value threshold: 10^–5^). GO terms and pathways enrichment analysis were performed with the hypergeometric test, and a Benjamini-Hochberg method corrected *P*-value ≤ 0.05 was considered to signify significantly enriched GO terms and pathways.

### Target MiRNAs and Genes Prediction, and Network Analysis

Pig miRNA sequences were obtained from the miRBase database and the binding sites of miRNA in circRNAs were predicted using miRanda (John et al., 2004) with a strict model. The 3′-untranslated region (UTR) sequences were downloaded from the UCSC Genome Browser^4^ and miRNA target gene predictions were performed using the miRanda with a strict model. The mRNA library data were derived from our previous study (Li et al., 2016) and the miRNA library data were deposited in NCBI SRA (Accession code: PRJNA606381; unpublished data) using the same samples (Bf28, Bf35, Bf36, Sol28, Sol35, and Sol36). The co-expression network of circRNA-miRNA-mRNA was constructed using DE circRNAs, miRNAs, and mRNAs and visualized using Cytoscape software (Shannon et al., 2003).

### qRT-PCR

Total RNA was extracted from the three Bf (Bf28, Bf35, and Bf36) and Sol (Sol28, Sol35, and Sol36) muscles used in the RNA-seq and reversed to complementary DNA (cDNA) with a Primescript RT Master Kit (Takara, Dalian, China) with random primers in accordance with the manufacturer’s instructions. Then, qRT-PCR was performed on a QuantStudio 7 Flex Real-Time PCR system (Thermo, Waltham, MA, United States) using AceQ qPCR SYBR Green Master Mix (Vazyme, Nanjing, China). The PCR condition was as follows: 5 min at 95°C for initial enzyme activation, followed by 10 s at 95°C for 40 amplification cycles, and then 30 s at 60°C. The divergent primer was designed as described previously (Panda and Gorospe, 2018). We first obtained the PCR amplicon template sequence by joining the 100–200 nt sequence from the 3′ end to the 100–200 nt sequence at the 5′ end of the circRNA. The PCR amplicon template sequence described above was then used to design the PCR primers using Primer 5 software. Primer sequences used in the qRT-PCR are listed in Supplementary Table S1. All reactions were performed in triplicate for each sample. The expression level of circRNAs was normalized to the reference gene glyceraldehyde-3-phosphate dehydrogenase (*GAPDH*), and the relative expression level of the circRNAs was calculated via the comparative CT method (2^–ΔΔCT^).

### Transfection and Dual-Luciferase Assay

The miR-499-5p mimics were purchased from GenePharma (Shanghai, China). For the dual-luciferase assay, sequences of ssc_circ_0001554, ssc_circ_0001573, and ssc_circ_0013564 containing a miR-499-5p binding site were synthesized by TSINGKE Company (Nanjing, China) and inserted into the *Nhe*I/*Sal*I site in the pmirGLO Dual-Luciferase report vector. HEK293T cell line was cultured in 24-well plates and co-transfected with 500 ng of the report vector and 15 pmol miR-499-5p mimics to each well using Lipofectamine^®^ 3000 reagent (Thermo Fisher Scientific, Waltham, MA, United States). At 24 h after transfection, luciferase activity was determined using a Glomax^®^ 20/20 luminometer platform (Promega, Madison, WI, United States) and a Double-Luciferase Reporter Assay Kit (Promega, Madison, WI, United States) following cells lysis. Transfections of each construct was conducted in triplicate, and firefly luciferase activity was normalized to the Renilla luciferase activity.

### Statistical Analysis

Statistical analyses of dual-luciferase assay and Pearson correlation coefficient were performed using SPSS 20.0 (SPSS Inc., Chicago, IL, United States). Results are expressed as the mean ± SEM, and statistically significant differences between two means were analyzed using Student’s *t*-test. A value of *P* < 0.05 was considered statistically significant.

## Results

### Identification and Characterization of CircRNAs in Bf and Sol Muscles

**FIGURE 1:**
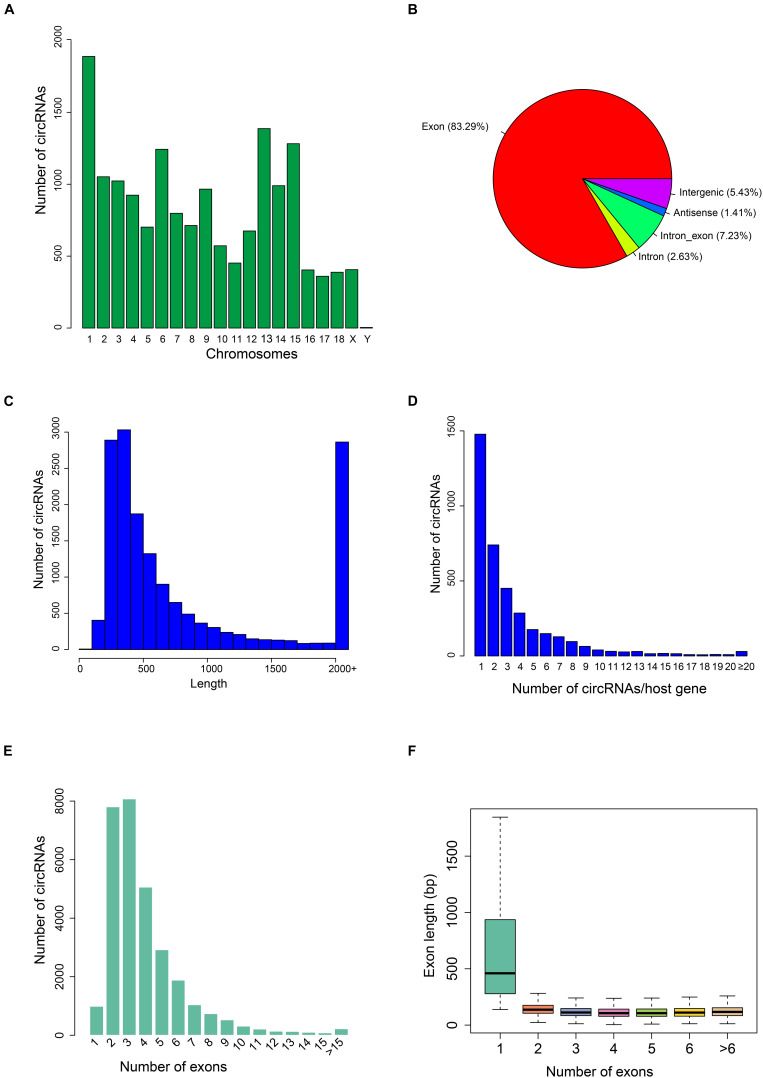
Characteristics of circRNAs in porcine Sol and Bf muscle tissues. **(A)** Distribution of the identified circRNAs in each chromosome. The *x*-axis represents the number of chromosomes and the *y*-axis represents the number of circRNAs classified by different chromosomes. **(B)** Classification of circRNAs in porcine skeletal muscle. **(C)** Length distribution of circRNAs. The *x*-axis represents the length of the identified circRNAs and the *y*-axis represents the abundance of (circRNAs classified by different lengths. **(D)** Distribution of the number of circRNA per gene. The *x*-axis represents the number of circRNAs/host gene and the *y*-axis represents the number of circRNA. **(E)** The exon number distribution for exon-derived circRNAs. The *x*-axis represents the number of exons that the circRNA contains and the *y*-axis represents the number of circRNA. **(F)** Box plot showing the exon length of exon-derived circRNAs. The *x*-axis represents the number of exons that the circRNA contains and the *y*-axis represents the exon length.)

To understand the expression characterization of circRNAs in fast-twitch Bf and slow-twitch Sol muscles, we sequenced ribosomal-depleted RNA in both types of muscle. First, we constructed six ribosomal-depleted RNA libraries from Bf and Sol muscles, which were denoted as Bf28, Bf35, Bf36, Sol28, Sol35, and Sol36 groups. We then performed RNA-seq for these libraries using a HiSeq Xten platform, from which 781,648,678 raw reads were obtained from the six libraries (Supplementary Table S2). Of these raw reads, 737,762,160 clean reads were yielded by removing the low-quality reads, polluted adapters, and reads with a high content of unknown base (N) content, as well as rRNA-mapped reads (Supplementary Table S2). The average quality value of Q30 (Phred quality score >30 and an error rate <0.001) was 94.60% for each library (Supplementary Table S2). Mapping results indicated that 736,890,033 of the clean reads were mapped to the porcine Sscrofa 11.1 reference genome (Supplementary Table S2). We also identified 16,342 high-confidence circRNAs from six ribosomal-depleted RNA libraries by at least two unique back-spliced reads (detailed summaries of all circRNAs are presented in Supplementary Table S3). Chromosomal distribution analysis indicated that circRNAs are widely distributed on all chromosomes, with the exception of the Y chromosome (Figure 1A); however, chromosome 1 and 13 contained more circRNAs than the other chromosomes (Figure 1A). Moreover, circRNAs were derived primarily from annotated exons, accounting for 83.29% of the entire circRNAs in Bf and Sol muscles (Figure 1B). Although the majority of the identified circRNAs were less than 1,000 nucleotides (nt) in length, many exceeded 2,000 nt (Figure 1C). We identified 16,342 circRNAs in the Bf and Sol muscles and predicted 3,809 host genes of these circRNAs (Supplementary Table S3). Notably, most host genes generate a single circRNA (Figure 1D). We then estimated that most circRNAs contain two to four exons (Figure 1E), but notably, the exon length of circRNAs consisting of only a single exon was longer than the circRNAs consisting of multiple exons (Figure 1F).

### DE CircRNAs Between Bf and Sol Muscle Types

**FIGURE 2:**
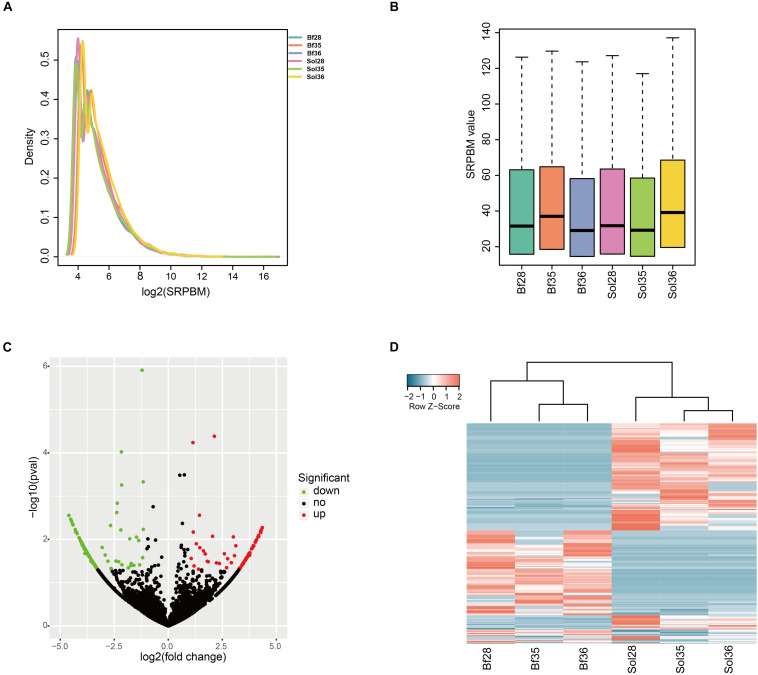
Expression analysis of circRNAs between Sol and Bf muscle tissues. **(A)** Density plot of the expression density distribution of circRNAs in each sample. **(B)** Box plot showing the expression abundance of circRNAs in each sample. **(C)** Volcano plot of all DE circRNAs between Sol and Bf tissues. The *x*-axis represents the value of log2 (Bf/Sol) and the *y*-axis represents the value of -log10 (*P*-value). Green, black, and red dots represent downregulated, unchanged, and upregulated DEcircRNAs, respectively, between the Sol and Bf groups. **(D)** Heatmap illustrating the relative expression of DE circRNAs from three Sol and three Bf tissues, with rows showing circRNAs and columns showing tissues.

To identify the key circRNAs regulating muscle fiber types, we estimated the expression level of, and performed differential expression analysis on, circRNAs in fast-twitch Bf and slow-twitch Sol muscles. We calculated the SRPBM value of all circRNAs identified in Bf and Sol muscles (detailed information is summarized in Supplementary Table S4). Results of analyses of the density and SRPBM distribution of circRNAs in each sample indicated that there were no significant differences between Bf and Sol muscles (Figures 2A,B). We further identified the DE circRNAs between Bf and Sol muscles. Among these DE circRNAs, 105 were upregulated and 137 were downregulated in Bf libraries compared to Sol libraries (Figure 2C; detailed summary of DE circRNAs is presented in Supplementary Table S5). The circRNA that was found to be most upregulated was ssc_circ_0005127, whereas ssc_circ_0006663 was the most downregulated (Supplementary Table S5). To further explore the expression patterns of DE circRNAs between the Bf and Sol muscle types, we performed a hierarchical clustering analysis and generated a gene expression heatmap of the DE circRNAs, the result of which indicated that these circRNAs have obvious differential expression patterns between the two muscle types (Figure 2D).

### Validation of DE CircRNAs Between Bf and Sol Muscles

**FIGURE 3:**
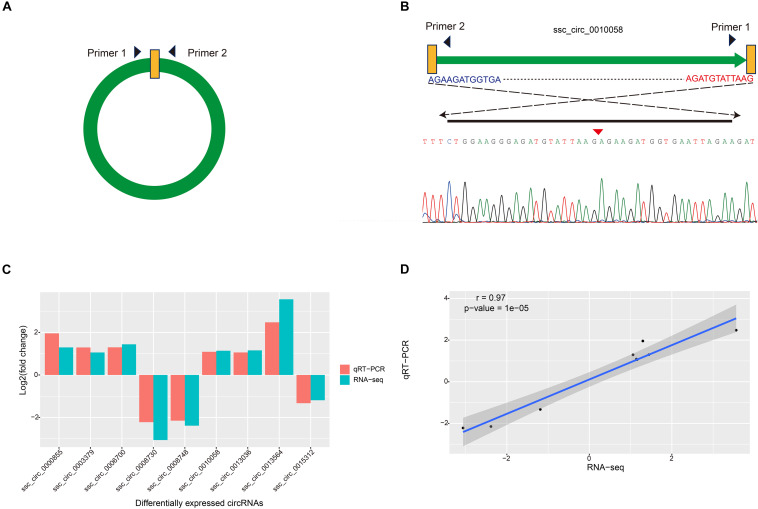
Validation of DE circRNAs by qRT-PCR. **(A)** Schematic of primer design for circRNAs used in the qRT-PCR. **(B)** Sanger sequencing was used to confirm the back-spliced junctions of circRNAs. **(C)** Validation and comparison of log2(fold change) in nine DE circRNAs between qRT-PCR and RNA-Seq. **(D)** Pearson correlation coefficient was calculated using comparing log2(fold change) data between qRT-PCR and RNA-Seq.

To verify the reliability of circRNAs identified in Bf and Sol muscles, expression levels of nine randomly chosen DE circRNAs were quantified via qRT-PCR. The divergent primers for each circRNA were designed based on the junction sequence (Figure 3A), and the expected size of PCR products was amplified using divergent primers for each circRNA; junction sequences for each circRNA were verified via Sanger sequencing (Figure 3B and Supplementary Figure S1). The results of this analysis indicated that expression patterns of these circRNAs as measured by qRT-PCR were consistent with the results obtained by RNA-seq (Figure 3C). Pearson correlation coefficient of the log2(fold change) data between the qRT-PCR and RNA-Seq was 0.97 (Figure 3D), suggesting that the DE circRNAs identified via RNA-seq were reliable.

### GO and Pathway Enrichment Analysis

**FIGURE 4:**
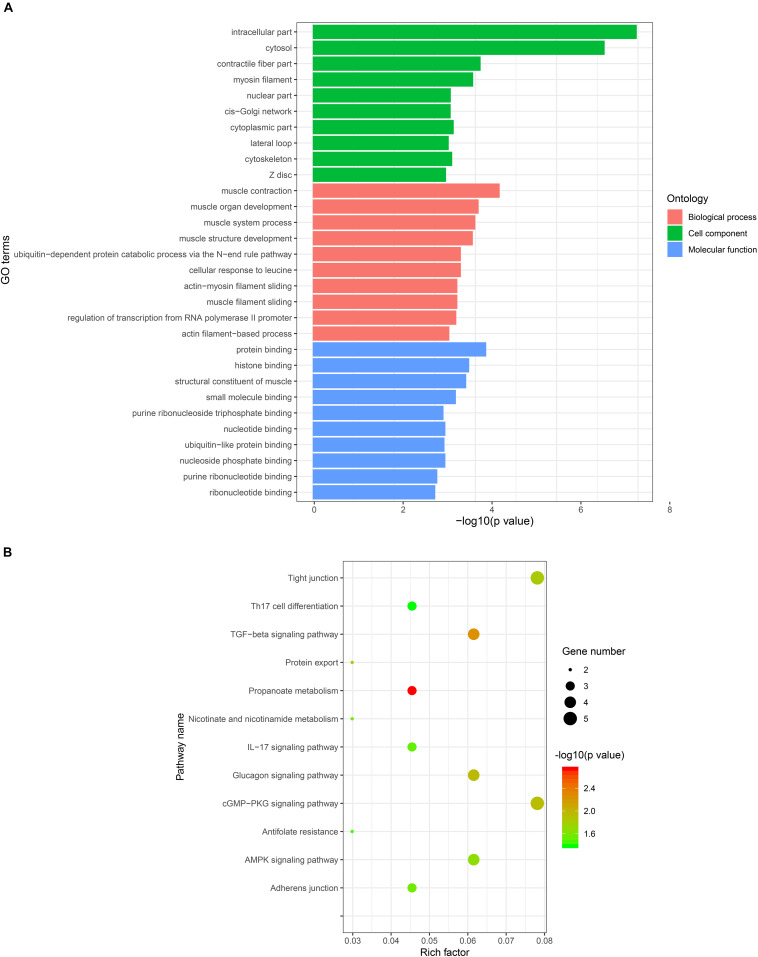
GO and KEGG enrichment analysis of host genes of DE circRNAs. **(A)** The most significantly enriched ten GO terms of DE circRNAs in biological process, cellular component, and molecular function. The *x*-axis represents GO terms and the *y*-axis represents the value of –log10 (*P*-value). **(B)** The most significantly enriched pathways of DE circRNAs. The *x*-axis represents a rich factor and the *y*-axis represents the pathway. The size and color of the bubble represent the number of genes enriched in the pathway and enrichment significance, respectively.

The results of a previous study have demonstrated that circRNAs regulate host gene transcription by competing with linear pre-mRNA splicing (Ashwal-Fluss et al., 2014). To explore the potential function of the host genes of DE circRNAs in the formation of skeletal muscle fiber, we performed GO and pathway enrichment analysis of host genes, which revealed that 347, 80, and 120 GO terms were significantly enriched in the biological process, cellular component, and molecular function, respectively (Supplementary Table S6). The ten most significant GO terms in the biological process, cellular component, and molecular function were shown in Figure 4A. Notably, host genes of DE circRNAs were found to be primarily involved in muscle biology, including muscle contraction, muscle organ development, muscle system process, muscle structure development, actin-myosin filament sliding, and muscle filament sliding in biological process; myosin filament, contractile fiber part, and Z disk in cellular component; structure constituent of muscle in molecular function (Figure 4A). Pathway enrichment analysis showed that the host genes were significantly enriched in 12 pathways (Supplementary Table S7), including the skeletal muscle fiber-related signaling pathways, such as the cGMP-PKG and AMPK signaling pathways (Figure 4B).

### Functional Analysis of CircRNA as a MiRNA Sponge

**FIGURE 5:**
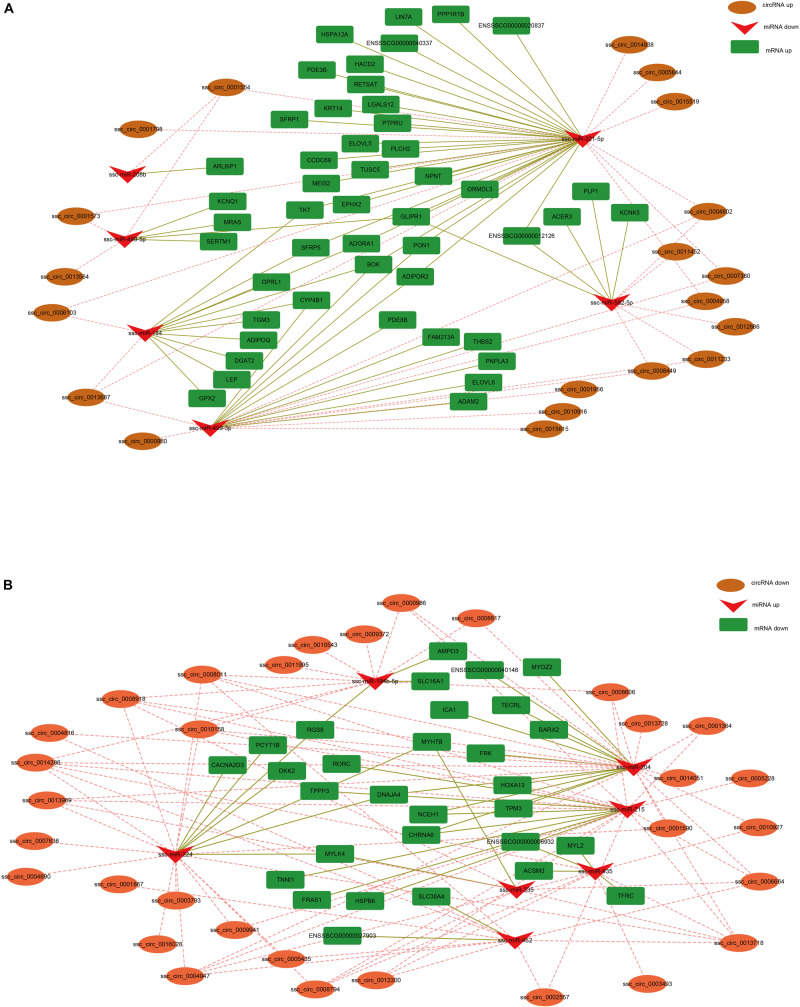
CircRNA-miRNA-mRNA regulatory network analysis. **(A)** Upregulated circRNA networks and **(B)** downregulated circRNAs networks.

Earlier studies have shown that circRNA can regulate gene expression by acting as a miRNA sponge (Hansen et al., 2013). To construct the co-expression network of circRNA-miRNA-mRNA, we integrated our previous mRNA (Li et al., 2016) and miRNA libraries data (unpublished data) to analyze the miRNA-binding sites in the circRNAs and mRNA using miRanda. In the up-down-up regulation pattern, we predicted 32 circRNA-miRNA and 59 miRNA-mRNA interactions (Figure 5A). As shown in Figure 5A, upregulated ssc_circ_0001554 may serve as a sponge for multiple miRNAs (ssc-miR-499-5p, ssc-miR-208b, and ssc-miR-221-5p). Notably, three circRNAs, including ssc_circ_0001573, ssc_circ_0001554 and ssc_circ_0013564, contained seed-targets of ssc-miR-499-5p, which were identified by searching for miRNA target sites. Moreover, these miRNAs can downregulate the expression of their target genes (i.e., ssc-miR-499-5p was predicted to bind with KCNQ1, MRAS, and SERTM1 genes). We further constructed a down-up-down co-expression network using miRanda with a strict model, for which 80 circRNA-miRNA and 36 miRNA-mRNA interactions were predicted (Figure 5B). In the down-up-down co-expression network, downregulated ssc_circ_0000986 were predicted to have three target microRNAs, (i.e., ssc-miR-194b-5p, ssc-miR-204 and ssc-miR-215). However, upregulated ssc-miR-215 was predicted to bind with multiple target genes, including NCEH1, CHRNA6, RORC, TPPP3, HSPB6, FRAS1, and TNNI1.

**FIGURE 6:**
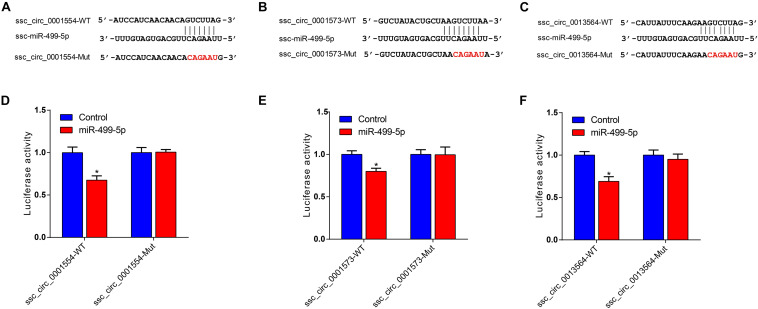
ssc_circ_0001573, ssc_circ_0001554 and ssc_circ_0013564 act as sponges for ssc-miR-499-5p. **(A–C)** The predicted binding site and mutated site of ssc-miR-499-5p in ssc_circ_0001573, ssc_circ_0001554, and ssc_circ_0013564. **(D–F)** Luciferase assay using reporter constructs with wild-type (WT) or mutant (Mut) of ssc_circ_0001573, ssc_circ_0001554, and ssc_circ_0013564. HEK293T cells in 24-well plates were transfected with the WT or Mut luciferase reporters of ssc_circ_0001573, ssc_circ_0001554 and ssc_circ_0013564, along with ssc-miR-499-5p mimics or control. Data are shown as the mean ± SEM (*n* = 3). *P < 0.05, two-tailed unpaired *t*-test.

Given that ssc_circ_0001573, ssc_circ_0001554, and ssc_circ_0013564 act as ssc-miR-499-5p sponges, we next investigated the binding capability of the ssc_circ_0001573, ssc_circ_0001554, and ssc_circ_0013564 to ssc-miR-499-5p using dual-luciferase assay. Figures 6A–C shows the predicted binding site and mutated site of ssc-miR-499-5p in ssc_circ_0001573, ssc_circ_0001554, and ssc_circ_0013564. These circRNAs dual-luciferase reporter vectors were constructed and co-transfected into HEK293T cells with the ssc-miR-499-5p mimics or control. The results showed that ssc-miR-499-5p significantly reduced the luciferase activity of wild type luciferase reporters of ssc_circ_0001573, ssc_circ_0001554, and ssc_circ_0013564 compared to control (Figures 6D–F), whereas, ssc-miR-499-5p had no effect on the mutated luciferase reporters. These results suggest that ssc_circ_0001573, ssc_circ_0001554, and ssc_circ_0013564 can all bind to and function as sponges for ssc-miR-499-5p.

## Discussion

Previous research has shown that circRNAs function as key post-transcriptional regulators in animals (Memczak et al., 2013). In this study, we identified 16,342 circRNAs from fast-twitch Bf and slow-twitch Sol muscles that may potentially play important role in skeletal muscle growth and development. Although a large number of circRNAs have been identified via RNA-seq in other studies involving pigs (Veno et al., 2015; Liang et al., 2017; Wang et al., 2019), the number of identified circRNAs in our study greatly exceeds that of earlier studies, and indication that the function of many circRNAs remains unknown, and that a greater number of circRNAs can be identified when sequencing depth is increased and analysis methodologies are improved. CircRNAs are produced via different mechanisms and have multiple origins, thus resulting in different forms (Dong et al., 2017; Li X. et al., 2018). The results of our analysis suggest that circRNAs are generated from diverse genomic locations (intergenic, exon, intron, and antisense) but derived primarily from exon circularization, findings consistent with those of previous studies involving a variety of different species, including human, mice, cattle, sheep, and pigs (Rybak-Wolf et al., 2015; Zheng et al., 2016; Legnini et al., 2017; Liang et al., 2017; Wei et al., 2017; Hao et al., 2019). As such, mammalian circRNAs may primarily be formed by precursor mRNA back-splicing of the exons. Moreover, the circRNAs identified in our analysis contain multiple exons, with most containing two or three exons, and this phenomenon has also been found in other studies (Zhang X. O. et al., 2014; Wei et al., 2017).

Previous research has shown that circRNAs are essential for proper functioning of many physiological and pathological processes, such as muscle development (Legnini et al., 2017), testes development (Hansen et al., 2013), cancer development, and innate immune responses (Li X. et al., 2018). Moreover, many circRNAs were known to exhibit distinct spatio-temporal expression patterns (Westholm et al., 2014; Szabo et al., 2015). Although circRNAs play important roles in muscle development (Li H. et al., 2018; Peng et al., 2019), their contribution to skeletal muscle fiber type formation remains unknown. Muscle fibers are classified into four types (type I, type IIa, type IIx, and type IIb) based on their particular physiological and metabolic properties. Type I muscle fibers, also known as slow-twitch muscle fibers, are rich in mitochondria and capillaries, exhibit oxidative metabolism, and have a low velocity of shorting and a high resistance to fatigue. Type II fibers, in contrast, are known as fast-twitch muscle fibers, and have comparatively fewer mitochondria and capillaries, exhibit fast-glycolytic capacity, and shrink faster and are easily fatigued (Bassel-Duby and Olson, 2006; Gundersen, 2011). Biceps femoris riched in type II muscle fiber is a typical fast-twitch muscle, whereas soleus (Sol) riched in type I muscle fiber is a typical slow-twitch muscle. As such, we used both fast-twitch Bf muscle and slow-twitch Sol muscle to identify which circRNAs control the development of skeletal muscle fiber types. We found that 242 DE circRNAs were identified between Bf and Sol muscles, and thus represent promising candidates for the regulation of skeletal muscle fiber transformation.

It has been proposed that circRNAs derived from exons of protein-coding genes and circularization of exons can compete with the splicing of their precursor transcripts (Ashwal-Fluss et al., 2014; Kelly et al., 2015). For example, Kelly et al. (2015) found that exon circularization attenuates the abundance of their linear mRNA expression levels, suggesting that circRNAs may affect biological function through regulation of linear mRNA expression levels. GO enrichment analysis of DE circRNA host genes revealed that many of these genes are involved in muscle biology, indicating that DE circRNAs may be essential for skeletal muscle growth and development. Moreover, multiple contraction metabolism-related GO terms, including muscle contraction, contractile fiber part, and Z disk, were significantly enriched, which suggests that DE circRNAs between Bf and Sol may regulate the transformation of muscle fiber type. In addition, KEGG analysis of DE circRNA host genes demonstrated that these genes were significantly enriched in the AMPK and cGMP-PKG signaling pathways; previous work has shown that AMPK signaling enhances oxidative metabolism and oxygen consumption rates (OCRs) in skeletal muscle (Witczak et al., 2008), and is essential for the conversion of glycolytic muscle fibers into oxidative fibers (Rockl et al., 2007; Lee et al., 2015). AMPK signaling can stimulate its downstream transcription factor PGC-1α, a gene that plays a crucial role in mitochondrial biogenesis, leading to the transformation of fast-to-slow muscle fibers (Lin et al., 2002), whereas the cGMP-PKG signaling pathway participates in muscle contraction (Kuo and Ehrlich, 2015). Skeletal muscles with different muscle fibers differ in their physiological and metabolic characteristics, such as glycolytic potential, oxidative metabolic capacity, and contractile properties (Gundersen, 2011). Bf and Sol muscle are skeletal muscles that contain different muscle fibers, leading us to speculate that DE circRNAs may regulate skeletal muscle fiber types conversion by mediating the AMPK and cGMP-PKG signaling pathways.

MicroRNAs (miRNAs), a class of small non-coding RNAs, can post-transcriptionally regulate gene expression by binding to their targets (Kim et al., 2006). Studies have shown that circRNAs function as competitive endogenous RNAs (ceRNAs) to bind miRNAs (Hansen et al., 2013; Huang et al., 2017; Yu et al., 2017). For example, ciRS-7 serves as a miR-7 sponge and strongly suppresses miR-7 activity, increasing the target expression levels of miR-7 (Hansen et al., 2013). CircHIPK2 influences astrocyte activation by regulating the autophagy and endoplasmic reticulum (ER) stress through its actions as a sponge of MIR124-2HG (Huang et al., 2017). In the present study, co-expression network analyses revealed that the DE circRNAs ssc_circ_0001573, ssc_circ_0001554, and ssc_circ_0013564 bind miR-499-5p. Intriguingly, miR-499-5p has been shown to play a crucial role in the conversion of skeletal muscle fibers by increasing oxidative fiber gene expression and repressing glycolytic fiber gene expression (van Rooij et al., 2009; Nachtigall et al., 2015; Horak et al., 2016; Xu et al., 2018). Moreover, analysis of expression profile indicated that ssc_circ_0001573, ssc_circ_0001554, and ssc_circ_0013564 are more highly expressed in the fast-twitch muscle than in slow-twitch muscle, the opposite of miR-499 expression pattern in the two muscle types. In addition, the results of the dual-luciferase assay showed that miR-499-5p can bind to ssc_circ_0001573, ssc_circ_0001554, and ssc_circ_0013564, and indication that these circRNAs may regulate skeletal muscle fiber transformation through interaction with miR-499-5p. Interaction network analyses indicated that ssc_circ_0001554 functions as a sponge of miR-208b, and previous studies have shown that miR-208b plays a pivotal role in the specification of muscle fiber through the activation and repressing of slow and fast myofiber gene programs, respectively (van Rooij et al., 2009), suggesting that ssc_circ_0001554 may regulate the conversion of slow- to fast-twitch muscle fibers by acting as a miR-208b sponge. Fiber-type switching may provide potential therapeutic targets for the treatment of metabolic and muscle diseases (Schiaffino et al., 2007), therefore, the results from the co-expression network analyses provides a possible mechanism regulating the conversion of skeletal muscle fiber in metabolic and muscle diseases, although the specific regulatory process is still needed to be confirmed.

We predicted that many circRNAs serve as ceRNAs to regulate fast and slow myofiber gene programs in the co-expression network. For example, fifteen upregulated circRNAs (including ssc_circ_0004947, ssc_circ_0010158, ssc_circ_0008794, ssc_circ_0008011, ssc_circ_0013969, ssc_ circ_0005228, ssc_circ_0001364, ssc_circ_0014286, ssc_circ_ 0002557, ssc_circ_0013718, ssc_circ_0006664, ssc_circ_0009941, ssc_circ_0008606, ssc_circ_0012300, and ssc_circ_0008918) in slow muscle fiber may regulate the expression level of the slow muscle gene *TNNI1* by sponging ssc-miR-215. Similarly, the ssc_circ_0004947, ssc_circ_0010158. and ssc_circ_0008011, were upregulated in the Sol muscle and may sponge ssc-miR-204 to regulate the level of the *MYOZ* gene. Given that the transformation of slow and fast muscle fiber was controlled by multiple slow and fast myofiber gene programs, respectively, so we speculated that these DE circRNAs function as a sponge for miRNAs that target fast or slow myofiber genes, thereby regulating the fast and slow muscle fiber transformation.

## Conclusion

We generated a comprehensive expression profile of circRNAs in fast-twitch Bf and slow-twitch Sol muscles and identified 16,342 circRNA candidates, which may benefit future investigations of circRNA regulatory mechanisms controlling skeletal muscle growth and development. Bf, a typical fast-twitch muscle, is riched in type II muscle fiber, whereas Sol riched in type I muscle fiber is a typical slow-twitch muscle. So, the 242 DE circRNAs identified between Bf and Sol muscle represent potential promising candidates for the circRNAs controlling the transformation of skeletal muscle fiber. We also constructed a co-expression network of DE circRNA-miRNA-mRNA, which provides a possible mechanism regulating the formation of skeletal muscle fiber. Because muscle fiber types are closely related to muscle diseases in humans and meat quality traits in livestock, the information obtained in this study about circRNA could be helpful in elucidating the genetic mechanisms underlying muscle diseases in human and meat quality traits in livestock.

## Data Availability Statement

Data are available from the corresponding author upon request. Transcriptome and miRNA sequence data have been deposited in NCBI SRA (accession codes PRJNA597666 and PRJNA606381, respectively).

## Ethics Statement

The animal study was reviewed and approved by the Nanjing Agricultural University. Written informed consent was obtained from the owners for the participation of their animals in this study.

## Author Contributions

WW conceived the study. BL, DY, PL, ZZ, and XZ conducted the experiments. HLi, LH, and RL analyzed the data. BL wrote the manuscript. HLiu and WW revised the manuscript.

## Conflict of Interest

The authors declare that the research was conducted in the absence of any commercial or financial relationships that could be construed as a potential conflict of interest.

## Footnotes

1 http://www.geneontology.org

2 http://www.blast2go.de/

3 http://www.genome.jp/kegg/

4 https://genome.ucsc.edu
