# Supplementary material for: Profiling and Functional Analysis of Circular RNAs in Porcine Fast and Slow Muscles
Source: Front Cell Dev Biol. 2020 May 26;8:322. doi: 10.3389/fcell.2020.00322 (PMC7264268; doi:10.3389/fcell.2020.00322)

Supplementary Figure S1 Sanger sequencing of circRNAs

ssc_circ_0000855

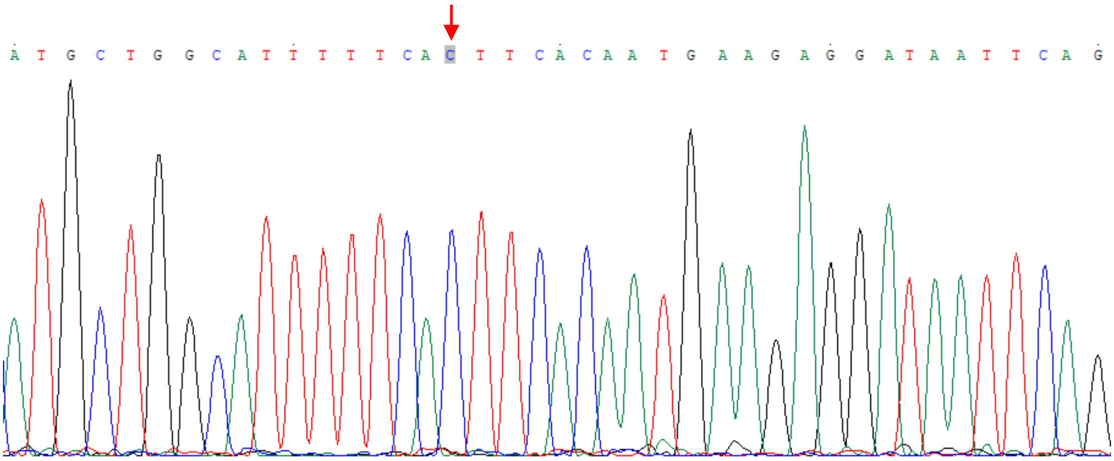

ssc_circ_0003379

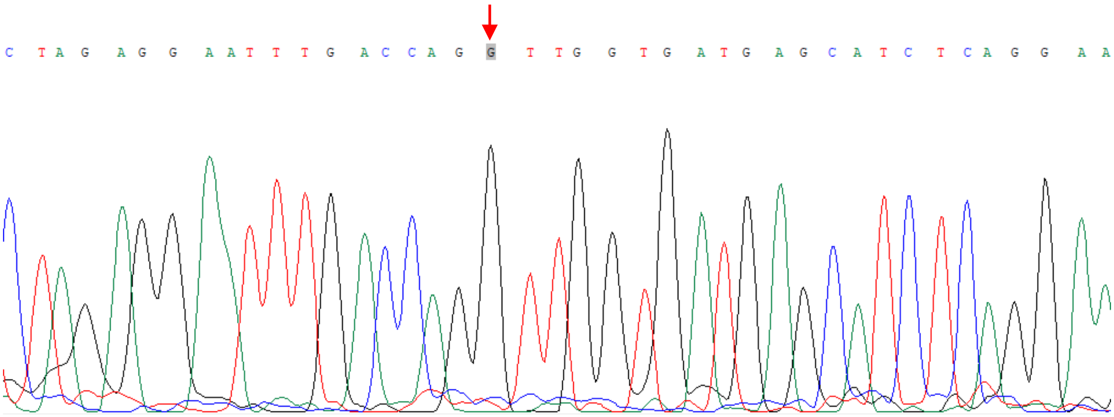

ssc_circ_0006700

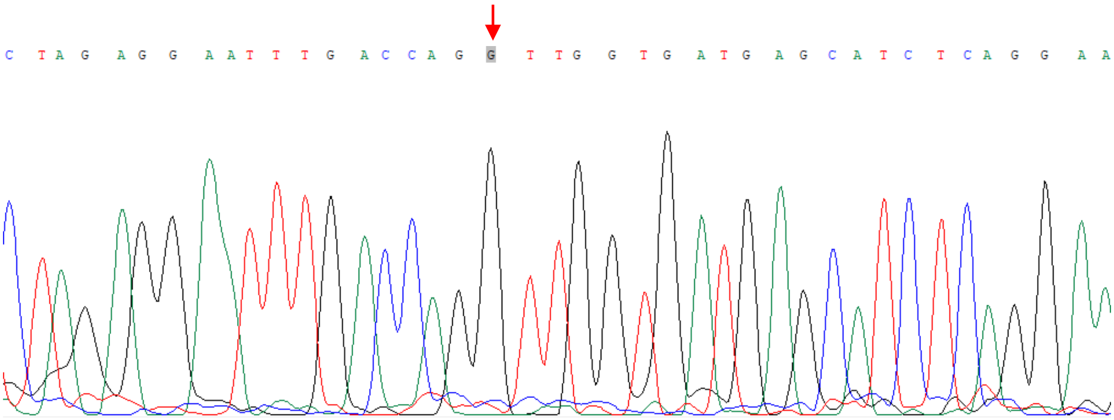

ssc_circ_0008730

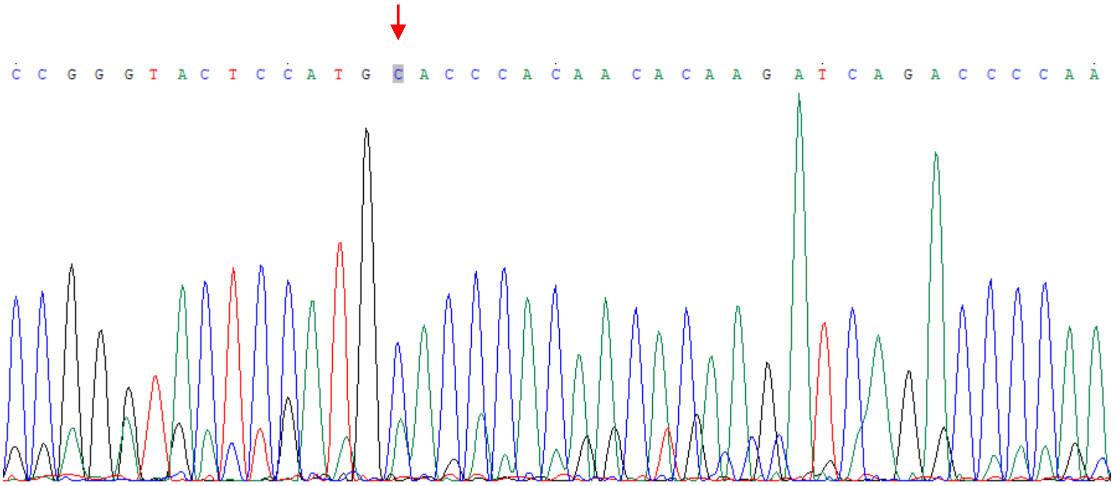

ssc_circ_0008748

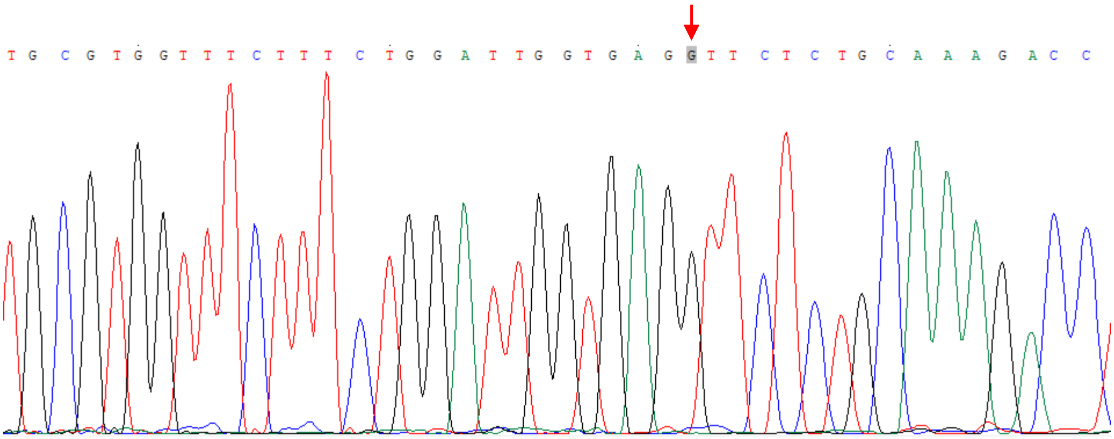

ssc_circ_0010058

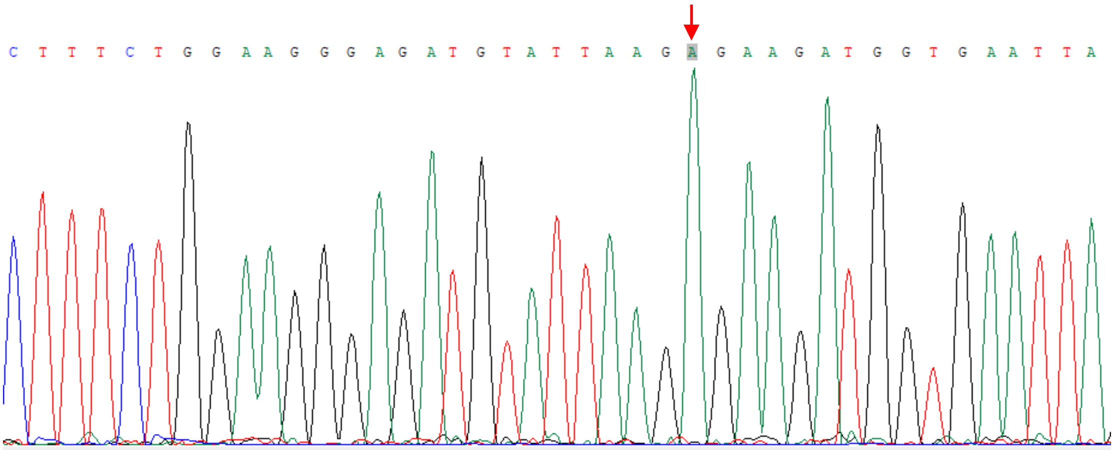

ssc_circ_0013036

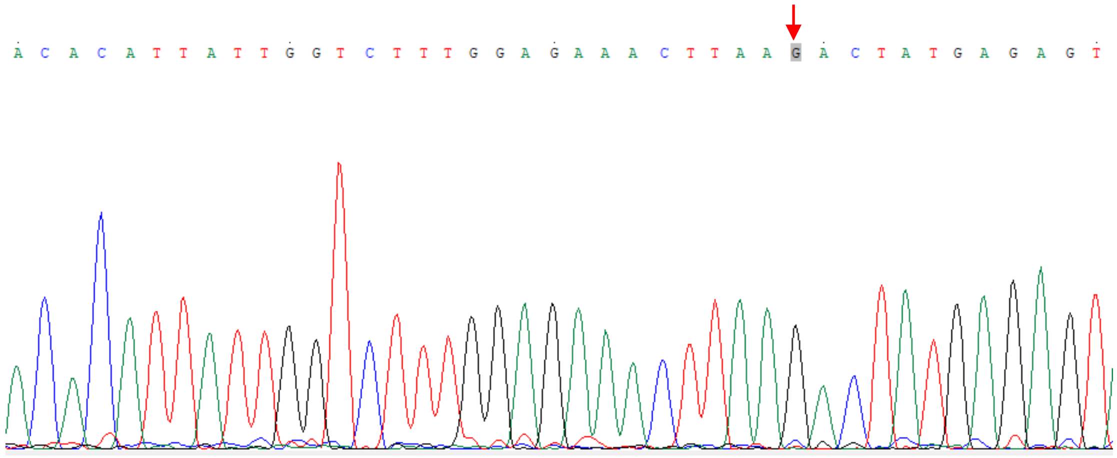

ssc_circ_0013564

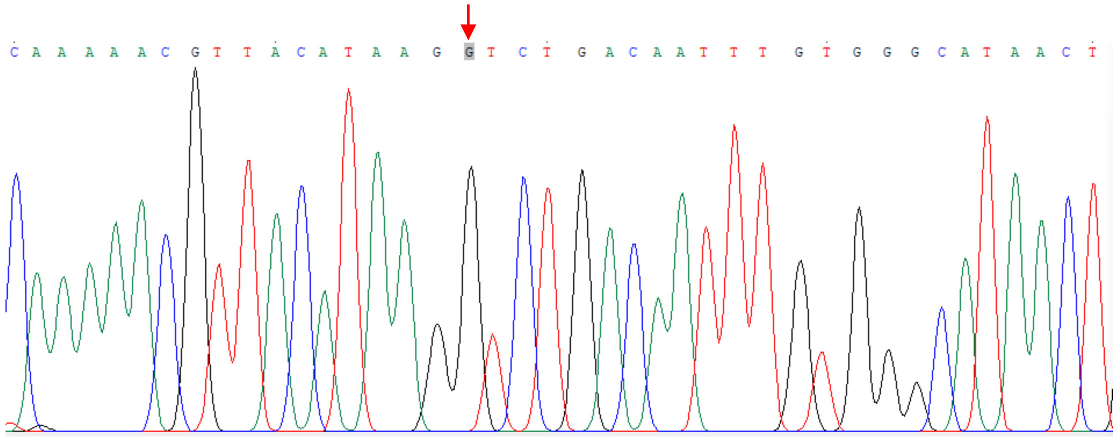

ssc_circ_0015312

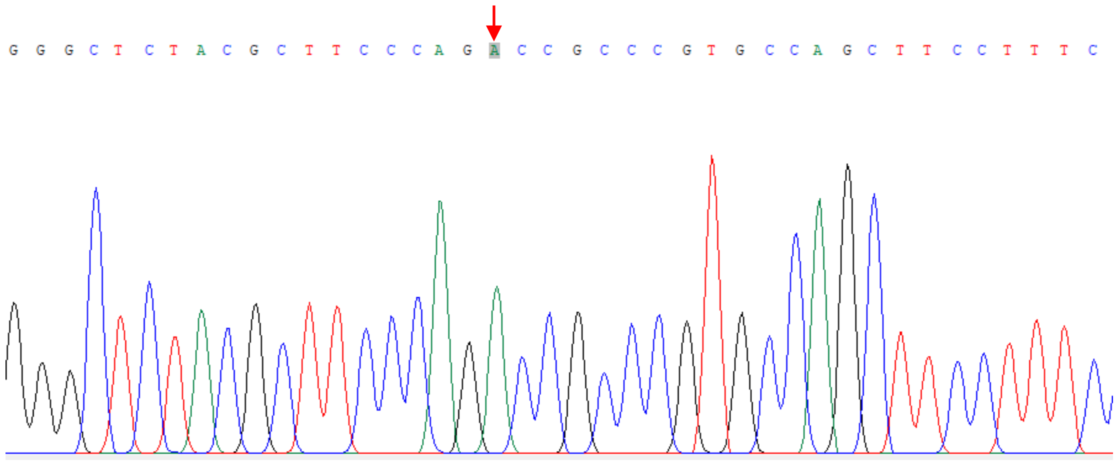

Supplement: Supplementary file 1 [file Data_Sheet_1.zip › Supplementary Materials/Supplementary Figure S1 Sanger sequencing of circRNAs.pdf]
