# Supplementary material for: Profiling and Functional Analysis of Circular RNAs in Porcine Fast and Slow Muscles
Source: Front Cell Dev Biol. 2020 May 26;8:322. doi: 10.3389/fcell.2020.00322 (PMC7264268; doi:10.3389/fcell.2020.00322)
Supplement: Supplementary file 1 [file Data_Sheet_1.zip › Supplementary Materials/Supplementary Table S1 Primers used in this study.docx]

**Supplementary Table S1 Primers used in this study**

| Gene name | Primer sequences | Product size | Annealing temperature |
| --- | --- | --- | --- |
| ssc_circ_0000855 | F: CAGCAGCAGAAACATCTCAAAG  R: GGAGAAGAGTCAGCCTCAAGTC | 239 bp | 60°C |
| ssc_circ_0003379 | F: GGGGCTCTTGACATAATGGTGA  R: TAGCATCCGGTGCTGCTTCCT | 117 bp | 60°C |
| ssc_circ_0006700 | F: GAAGCATCCTCAGCCACAAC  R: TTCAGTAAGGGTCCAACACC | 199 bp | 60°C |
| ssc_circ_0008730 | F: AGCCTCTGCCAATGGAGAC  R: CAGGAAGGGGAACACGAAA | 376 bp | 60°C |
| ssc_circ_0008748 | F: TGCTGCGTGGTTTCTTTCT  R: CACTGGCTCCTGCTGGCTA | 104 bp | 60°C |
| ssc_circ_0010058 | F: CTTTCAAGAGTACAGCAAACCAG  R: AATCCTGCATCATCTATTTCACC | 172 bp | 60°C |
| ssc_circ_0013036 | F: GATCTGAAGAATGGAGCAACACAT  R: CCAGAGTAAGTCCCTTAACGAGGT | 233 bp | 60°C |
| ssc_circ_0013564 | F: GCTGAAGTCGAAACACGGATT  R: GCTTGAACGAGACTGTGGAGGT | 254 bp | 60°C |
| ssc_circ_0015312 | F: TGGTCAAGTCTACCACCAAGCG  R: GAGGAGTTCTGGGAGGACATCG | 261 bp | 60°C |
| GAPDH | F: ATCACTGCCACCCAGAAGACT  R: CATGCCAGTGAGCTTCCCGTT | 153 bp | 60°C |
